# Symptomatic Tendon Sheath Ganglion at the Insertion of the Popliteus Tendon Treated With Local Cortisone Injection

**DOI:** 10.1155/cro/6686698

**Published:** 2026-03-11

**Authors:** Monika Reden, Jules-Nikolaus Rippke, George-Mihai Avram, Elias Ammann, Laszlo Toth, Matthias Koch, Randa Elsheikh, Michael Hirschman, Natalie Mengis

**Affiliations:** ^1^ Landesklinikum Baden-Mödling, Mödling, Niederösterreich, Austria, lknoe.at; ^2^ Orthopedics & Traumatology, Kantonsspital Baselland, Liestal, Basel-Landschaft, Switzerland, ksbl.ch; ^3^ Department of Clinical Research, Research Group Michael T. Hirschmann, Regenerative Medicine and Biomechanics, University of Basel, Basel, Switzerland, unibas.ch

## Abstract

Popliteus tendon sheath ganglia represent a rare and frequently overlooked cause of persistent lateral knee pain. This case report describes a patient with chronic symptoms unresponsive to conservative treatment, in whom advanced imaging revealed a ganglion at the lateral insertion of the popliteus tendon. Ultrasound‐guided corticosteroid injection resulted in complete resolution of symptoms. The case highlights the importance of thorough differential diagnosis using advanced imaging and supports the effectiveness of targeted, minimally invasive interventions. Informed consent for publication was obtained.

## 1. Introduction

The popliteus tendon is an important stabilizing structure of the knee with complex anatomical and functional characteristics. It originates from the lateral femoral condyle and inserts on the posterior surface of the tibia above the linea solea, coursing deep to the lateral collateral ligament through the popliteal hiatus. Although intracapsular, the tendon remains extra‐synovial. Anatomically, it can be divided into a proximal femoral origin; a middle portion with attachments to the posterior joint capsule, lateral meniscus, and proximal fibula via the popliteofibular ligament; and a distal insertion on the posterior tibia. Through its popliteomeniscal fascicles, the tendon contributes to stabilization of the lateral meniscus and interacts closely with the meniscofemoral ligaments and the posterior cruciate ligament [1, 2].

Functionally, the popliteus tendon acts as both a static and dynamic stabilizer of the knee. It facilitates internal rotation of the tibia in non‐weight‐bearing conditions and external rotation of the femur during weight bearing, playing a key role in the screw‐home mechanism and in unlocking the knee from terminal extension [3–5]. With increasing knee flexion, the tendon limits external rotation and posterior translation of the tibia while retracting the lateral meniscus posteriorly. Variability in tendon volume and physiological cross‐sectional area has been reported, further underscoring its relevance for dynamic knee stability [2, 3].

Clinically, injuries of the popliteus tendon are most commonly associated with varus stress, hyperextension, or forced external rotation of the tibia and frequently occur in conjunction with posterolateral corner and posterior cruciate ligament injuries [5–7]. Degenerative changes and calcification of the tendon may result in pain, swelling, and functional limitation and can be managed conservatively or surgically [8]. During knee arthroplasty, improper positioning or lateral overhang of the tibial component may cause iatrogenic tendon impingement, which has been reported as a source of persistent postoperative pain. Awareness of the anatomical course and preservation of the popliteus tendon are therefore essential to avoid postoperative complications [5, 9].

## 2. Case Report

**Figure 1:**
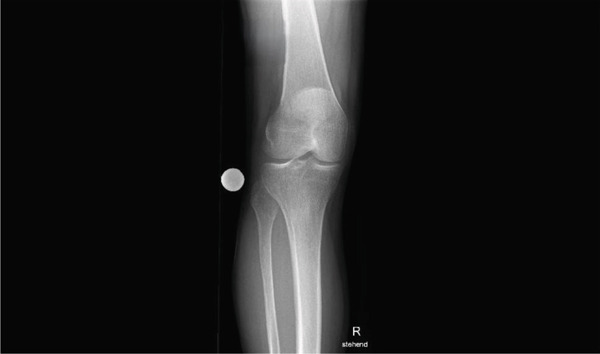
X‐ray of the right knee joint a.p. (anteroposterior) showing no abnormalities apart from early‐stage medial osteoarthritis. The letter "R" stands for the word "right," indicating that it is an imaging of a right limb.

**Figure 2:**
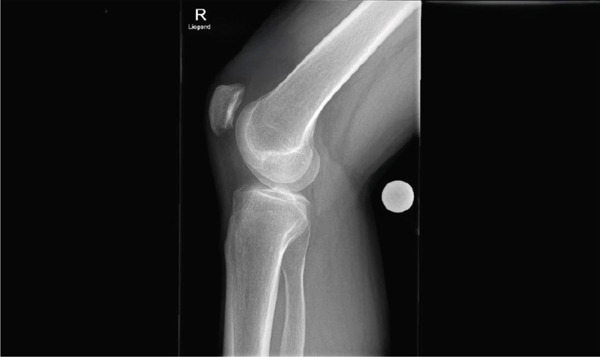
X‐ray of the right knee joint (lateral view). The letter "R" stands for the word "right," indicating that it is an imaging of a right limb.

**Figure 3:**
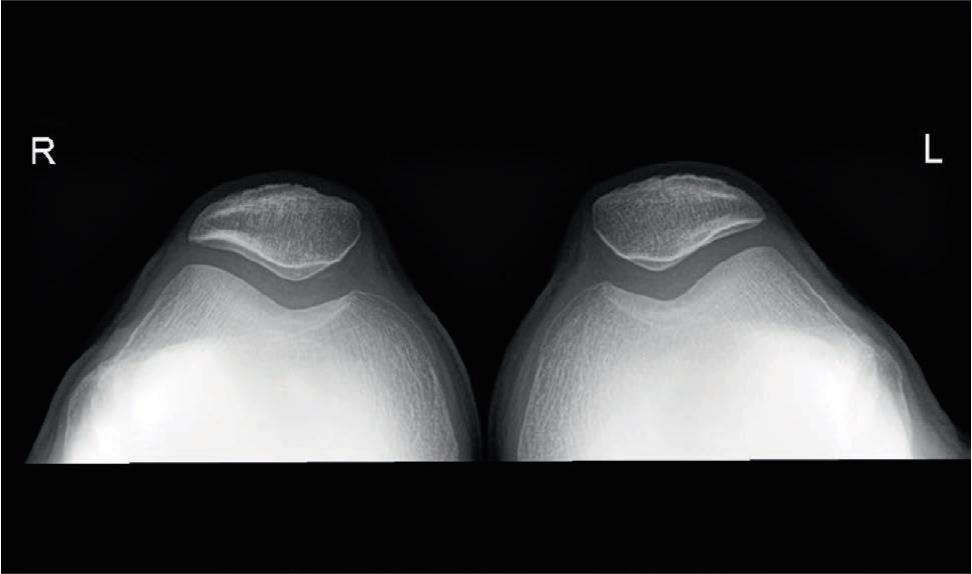
X‐ray of both patellae (axial view). The letter "R" stands for the word "Right", indicating that it is an imaging of a right limb. Accordingly the "L" stands for "Left."

A 47‐year‐old patient, employed as a service coordinator in an office setting, was referred to the hospital by his general practitioner for corticosteroid infiltration due to lateral right‐sided knee pain persisting for several months. The patient reported no history of trauma and did not engage in regular sports activities. Previous treatments, including shockwave therapy and fascial training, had not led to any improvement. In July 2023, an MRI of the knee joint had already been performed, revealing a nonspecific posteromedial capsular edema with isolated ganglia along the lateral insertion of the popliteus tendon. When the patient presented to the clinic for the first time in November 2023, an additional X‐ray of the knee joint was obtained (Figures 1, 2, and 3), which showed no abnormalities apart from early‐stage medial osteoarthritis. The patient was prescribed nonsteroidals such as ibuprofen for pain management. Additionally, he was referred to physiotherapy for evaluation, muscle relaxation, and trigger point massage.

**Figure 4:**
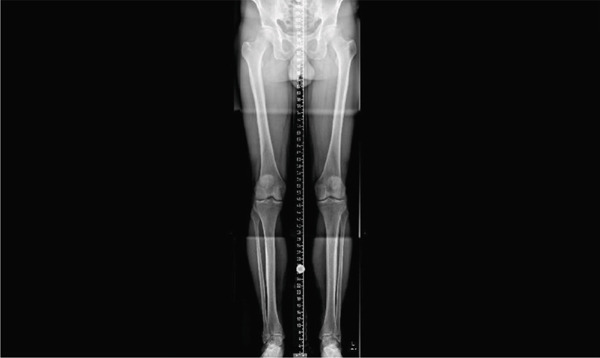
Full‐leg X‐ray.

At the clinical follow‐up in January 2024, the patient reported only minimal and temporary symptom relief. He described that the discomfort worsened during deep knee flexion followed by standing up, characterizing it as a pressure sensation resembling a “balloon bursting.” Subsequently, a full‐leg standing radiograph (Figure 4) was performed, along with a targeted physiotherapeutic evaluation, including a comprehensive assessment and the execution of functional testing.

## 3. Clinical Findings

During the clinical examination, the patient presented with a straight leg axis and a physiological hindfoot valgus, which shifted into varus during tiptoe standing. Pain was provoked at the level of the fibular head when rising from a deep squat, despite stability of the proximal tibiofibular joint. There was no tenderness along the iliotibial tract or at Gerdy′s tubercle. The patient described the pain as “paralyzing,” with a visual analog scale (VAS) score of 8–9. He reported pain aggravation during deep flexion (> 120°) and under loading. The patient demonstrated normal posture and gait. Tenderness over the fibular head persisted, but no aggravating or alleviating factors for the pain were identified. Additionally, the patient described a stretching pain during ankle dorsiflexion and supination, which he attributed to a history of a supination trauma 2 years prior. Functional testing of the knee joint identified deep squats as the primary problematic movement.

## 4. Diagnosis

Following the second physiotherapy evaluation, another follow‐up was conducted in February 2024, during which the results of the initial MRI diagnostics were reviewed with the patient. It was decided to perform a repeat MRI with contrast enhancement to exclude the possibility of pigmented villonodular synovitis (PVNS) and to reassess the visible irritation of the popliteus tendon. The imaging was conducted in the same month.

**Figure 5:**
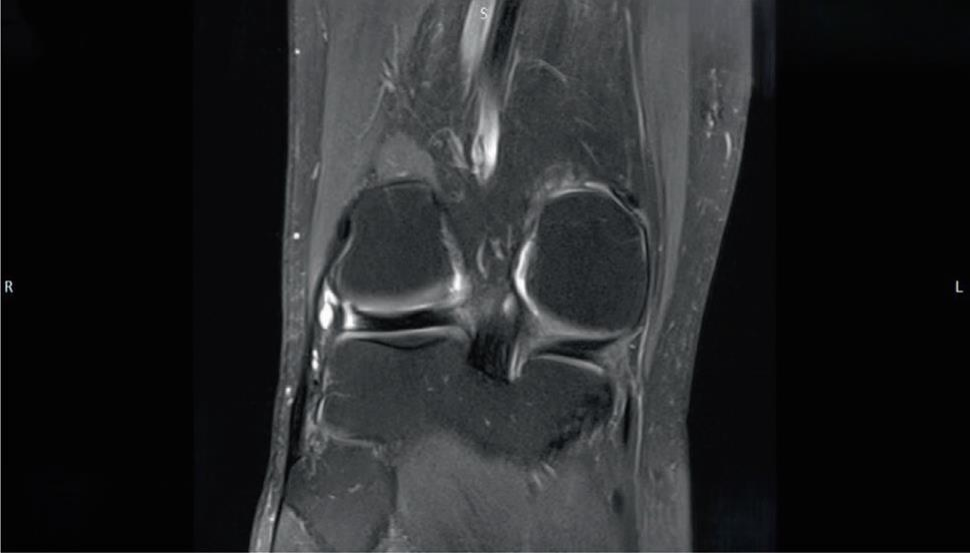
MRI of the right knee joint (coronal view) showing a stable ganglion at the origin and along the course of the popliteus tendon, with a point of maximum prominence at the level of the lateral femorotibial joint line. In this imaging the "R" stands for "right side" and the "L" stands for "left side."

**Figure 6:**
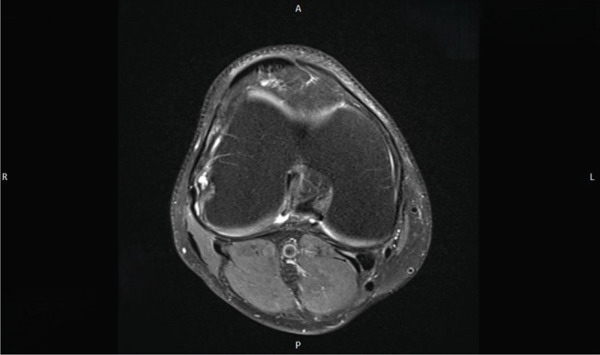
MRI of the right knee joint (axial view). In this imaging the "R" stands for "right side" and the "L" stands for "left side."

**Figure 7:**
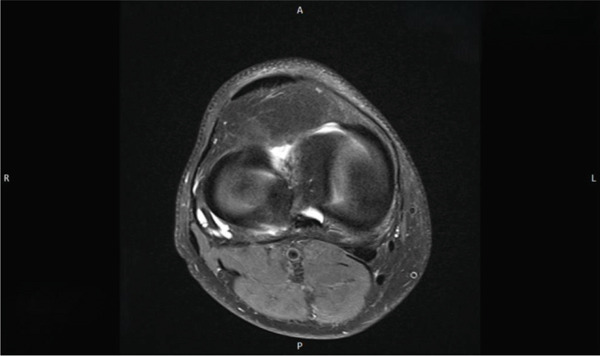
MRI of the right knee joint (axial view). In this imaging the "R" stands for "right side" and the "L" stands for "left side." Accordingly the letter "A" stands for "anterior" and the letter "P" stands for "posterior."

The contrast‐enhanced MRI (Figures 5, 6, and 7) revealed no evidence of hemosiderin deposits, thereby excluding the presence of a localized or diffuse tenosynovial giant cell tumor (PVNS). Additionally, a stable ganglion formation was observed at the origin and along the course of the popliteus tendon, with a point of maximum prominence at the level of the lateral femorotibial joint line. Incidental findings included a preexisting small subchondral edema at the lateral patellar facet, mucoid degeneration of the anterior cruciate ligament (ACL) with a small ganglion formation at its origin, and perifocal contrast enhancement at this site.

## 5. Treatment

**Figure 8:**
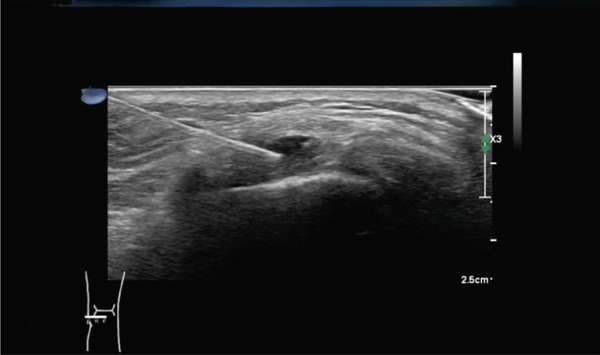
Ultrasound‐guided cortisone infiltration showing a multilobulated ganglion along the proximal course of the popliteus tendon, most likely corresponding to a tendon sheath ganglion.

Given the findings of the contrast‐enhanced MRI and the lack of symptom improvement, the indication was set to perform a local cortisone infiltration with one ampoule Rapidocain 2% and 40 mg Kenacort directly around the popliteus tendon at its proximal insertion on the fibular head. This indication was established because the MRI showed no associated pathology, and infiltration represents a targeted, noninvasive therapeutic option with anti‐inflammatory effects that may reduce symptoms and therefore prevent the need for surgical intervention. The procedure was thoroughly discussed with the patient, who ultimately provided consent. Subsequently, the ultrasound‐guided infiltration was scheduled for the 12th of March 2024. During the ultrasound assessment (Figure 8) performed at the time of infiltration, the attending radiologist described a multilobulated ganglion along the proximal course of the popliteus tendon, most likely corresponding to a tendon sheath ganglion.

At the clinical follow‐up 3 weeks after the infiltration in April 2024, the patient presented with a positive healing trajectory and was symptom‐free. Physiotherapy was prescribed again to address a slight muscular imbalance around the knee joint and to conclude the treatment. Further follow‐up would only be necessary if symptoms were to recur. Since the last follow‐up almost 1 year ago, the patient has not returned for a follow‐up.

## 6. Discussion and Limitations

Popliteus tendon ganglia are a rare and often underrecognized cause of lateral knee pain, typically associated with trauma or athletic activity as described in the case report of Vahabi et al. in 2023 and Blake et al. in 2005 [7, 10]. In contrast, the present case involved a nonathletic patient without prior trauma. A case report from Davalos et al. in 2016 also showed two cases of nonathletic patients without prior trauma, who also experienced gradually increasing lateral knee pain. The first case presented a split tear of the popliteus tendon at its proximal attachment on the lateral femoral condyle with surrounding soft tissue edema, while the second case presented a medially displaced popliteus tendon lodged within the lateral intercondylar space—an abnormal path which was not previously reported [11]. The purpose of this case report is to highlight the importance of including this pathology in the differential diagnosis even in atypical presentations (nonathletic and no prior trauma) to prevent misdiagnosing and further unnecessary and potentially harmful treatment. The popliteus tendon, with its complex anatomy and dual static dynamic stabilizing role in the knee joint, plays a crucial role in posterolateral stability of the knee [2, 3, 5]. Initial clinical findings in such cases may be nonspecific, and standard diagnostic approaches often fail to identify the underlying pathology. Our patient′s persistent pain despite conservative treatment warranted further imaging, which revealed a multilobulated ganglion adjacent to the popliteus tendon insertion. This mirrors the findings of Jose et al., who also described successful identification of a small popliteus tendon ganglion on MRI and ultrasound [12]. As in their report, ultrasound played a dual role in confirming the diagnosis and guiding the minimally invasive treatment. The successful outcome following ultrasound‐guided corticosteroid injection aligns with previous case‐based evidence supporting this as an effective first‐line treatment for tendon sheath ganglia [1, 12]. By directly targeting the lesion, the injection likely reduced inflammation and mechanical irritation, contributing to rapid symptom resolution. While surgical options such as arthroscopic removal have been described in cases of calcific tendinopathy [8], image‐guided injection presents a lower risk, outpatient‐friendly alternative with favorable results in selected cases. Biomechanical stress at the lateral insertion of the popliteus tendon may contribute to ganglion formation. Prior research indicates that repetitive strain, particularly during external tibial rotation and varus stress, can predispose to microtrauma in this area [5]. Although our patient′s coexisting findings, such as patellar maltracking and ACL mucoid degeneration, were not directly linked, they may reflect altered joint mechanics contributing to localized stress. Addressing these through physiotherapy can play a role in recurrence prevention. This case reinforces the importance of a multimodal, interdisciplinary treatment approach. Collaboration between orthopedics, radiology, and physiotherapy enabled precise diagnosis and individualized therapy. Continued rehabilitation post‐injection focused on correcting muscular imbalances and optimizing knee biomechanics, essential for preventing recurrence—a recommendation echoed in previous reports on pathologies of the popliteus tendon [3, 5]. In summary, tendon sheath ganglia of the popliteus tendon, though rare, should be considered in cases of unexplained lateral knee pain, particularly when conservative treatment fails. MRI and ultrasound are indispensable tools for identifying such lesions, and ultrasound‐guided corticosteroid injection represents a safe and effective therapeutic option. Interdisciplinary management and long‐term rehabilitation are crucial for sustained recovery. While current knowledge is largely based on isolated case reports [10, 12], further studies are needed to better understand the etiology, recurrence risk, and optimal treatment protocols for this underdiagnosed condition.

This case report has several inherent limitations that should be acknowledged. This report is limited by its single‐patient design, which precludes generalization of the findings. Although MRI and ultrasound findings were consistent with a tendon sheath ganglion, no histological confirmation was obtained. Symptom resolution followed corticosteroid injection without aspiration, making it difficult to determine the exact mechanism of improvement. In addition, follow‐up was based on clinical assessment without post‐interventional imaging, and longer term follow‐up would be required to evaluate recurrence risk.

## 7. Conclusions

The popliteus tendon should be recognized as a potential source of lateral knee pain, particularly in the absence of traumatic injury and when conservative treatments prove ineffective. Accurate diagnosis relies on modern imaging modalities, including contrast‐enhanced MRI and ultrasound, which help identify structural pathologies such as ganglia or tendinopathies. Effective management benefits from close interdisciplinary collaboration and a tailored therapeutic approach. Combining targeted physiotherapy with interventional procedures, such as ultrasound‐guided infiltration, followed by structured rehabilitation, appears to offer sustained symptom relief and functional recovery. Early intervention and continued physiotherapeutic care are essential to optimize knee biomechanics and prevent recurrence.

## Funding

No funding was received for this manuscript. Open access publishing facilitated by Universitat Basel, as part of the Wiley ‐ Universitat Basel agreement via the Consortium Of Swiss Academic Libraries.

## Conflicts of Interest

The authors declare no conflicts of interest.

## Data Availability

The data that support the findings of this study are available on request from the corresponding author. The data are not publicly available due to privacy or ethical restrictions.
